# Crystal structure of 3-methyl-1-phenyl-5-(1*H*-pyrrol-1-yl)-1*H*-pyrazole-4-carbaldehyde

**DOI:** 10.1107/S1600536814020984

**Published:** 2014-09-27

**Authors:** Joel T. Mague, Shaaban K. Mohamed, Mehmet Akkurt, Talaat I. El-Emary, Mustafa R. Albayati

**Affiliations:** a Department of Chemistry, Tulane University, New Orleans, LA 70118, USA; b Chemistry and Environmental Division, Manchester Metropolitan University, Manchester, M1 5GD, England; c Chemistry Department, Faculty of Science, Minia University, 61519 El-Minia, Egypt; d Department of Physics, Faculty of Sciences, Erciyes University, 38039 Kayseri, Turkey; e Department of Chemistry, Faculty of Science, Assiut University, 71515 Assiut, Egypt; f Kirkuk University, College of Science, Department of Chemistry, Kirkuk, Iraq

**Keywords:** crystal structure, pyrazole ring, pyrrolyl ring, dimers

## Abstract

In the title compound, C_15_H_13_N_3_O, the pyrrolyl and phenyl rings make dihedral angles of 58.99 (5) and 34.95 (5)°, respectively, with the central pyrazole ring. In the crystal, weak, pairwise C—H⋯O inter­actions across centers of symmetry form dimers, which are further associated into corrugated sheets running approximately parallel to (100) *via* weak C—H⋯N inter­actions.

## Related literature   

For the biological activity of pyrazoline-containing compounds see: Nauduri & Reddy (1998 ▶); Korgaokar *et al.* (1996 ▶); Taylor & Patel (1992 ▶); Ozdemir *et al.* (2007 ▶); Ruhoğlu *et al.* (2005 ▶); Palaska *et al.* (2001 ▶); Rajendra Prasad *et al.* (2005 ▶); Udupi *et al.* (1998 ▶). For synthetic and industrial applications of pyrazolo­[3,4-*b*]pyrazines see: Rangnekar & Dhamnaskar (1990 ▶); Kopp *et al.* (2001 ▶); Farghaly & El-Kashef (2005 ▶).
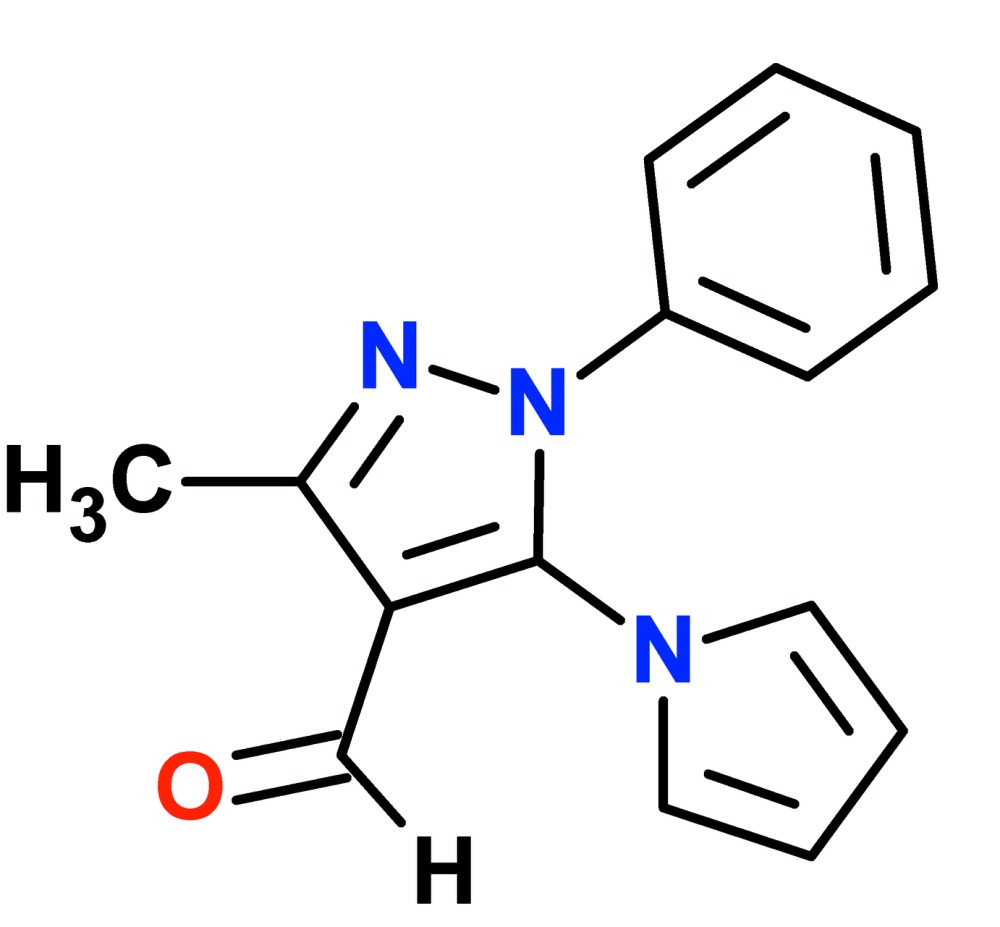


## Experimental   

### Crystal data  

C_15_H_13_N_3_O
*M* *_r_* = 251.28
Monoclinic, 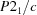

*a* = 9.5807 (8) Å
*b* = 15.1720 (13) Å
*c* = 8.7370 (8) Å
β = 93.6180 (11)°
*V* = 1267.46 (19) Å^3^
*Z* = 4
Mo *K*α radiation
μ = 0.09 mm^−1^
*T* = 150 K
0.29 × 0.17 × 0.04 mm

### Data collection  

Bruker SMART APEX CCD diffractometer
Absorption correction: multi-scan (*SADABS*; Bruker, 2014 ▶) *T*_min_ = 0.98, *T*_max_ = 1.00
26282 measured reflections
3321 independent reflections
2527 reflections with *I* > 2σ(*I*)
*R*_int_ = 0.053

### Refinement  

*R*[*F* ^2^ > 2σ(*F* ^2^)] = 0.044
*wR*(*F* ^2^) = 0.114
*S* = 1.07
3321 reflections
173 parameters
H-atom parameters constrained
Δρ_max_ = 0.31 e Å^−3^
Δρ_min_ = −0.25 e Å^−3^

### 

Data collection: *APEX2* (Bruker, 2014 ▶); cell refinement: *SAINT* (Bruker, 2014 ▶); data reduction: *SAINT*; program(s) used to solve structure: *SHELXT* (Bruker, 2014 ▶); program(s) used to refine structure: *SHELXL2014* (Sheldrick, 2008 ▶); molecular graphics: *DIAMOND* (Brandenburg & Putz, 2012 ▶); software used to prepare material for publication: *SHELXTL* (Sheldrick, 2008 ▶).

## Supplementary Material

Crystal structure: contains datablock(s) global, I. DOI: 10.1107/S1600536814020984/sj5428sup1.cif

Structure factors: contains datablock(s) I. DOI: 10.1107/S1600536814020984/sj5428Isup2.hkl

Supporting information file. DOI: 10.1107/S1600536814020984/sj5428Isup3.cml

**Fig. 1.**
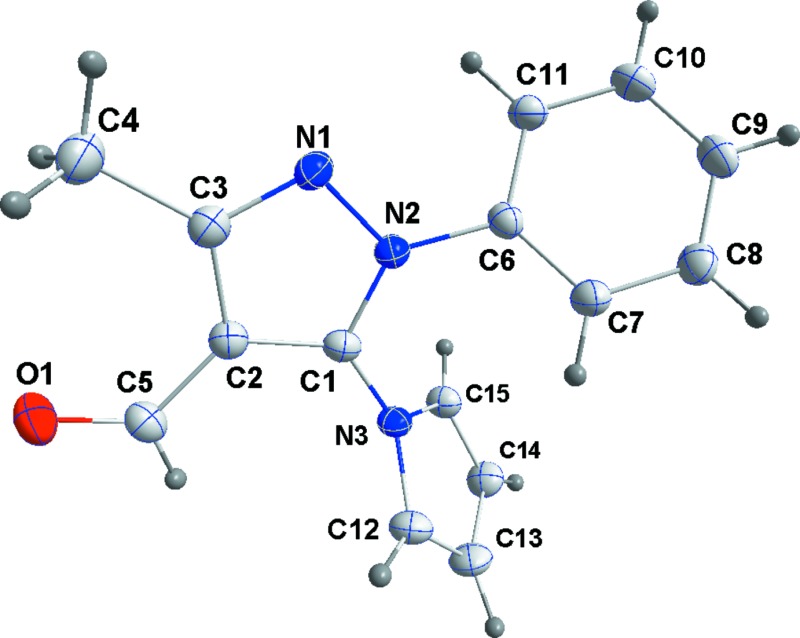
Perspective view of the title molecule with labeling scheme and 50% probability ellipsoids for non-H atoms.

**Fig. 2.**
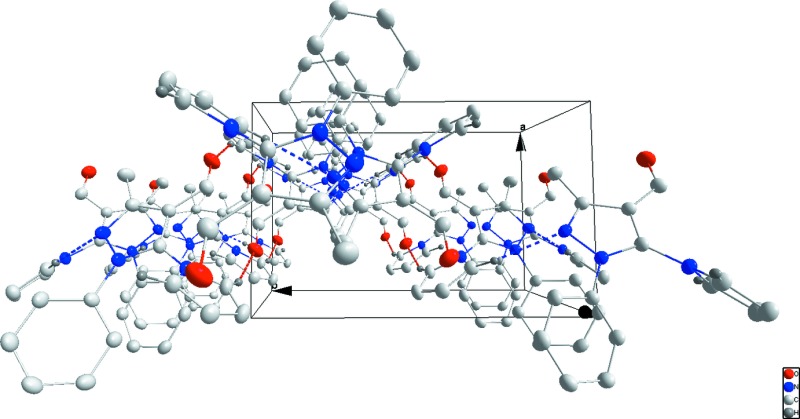
Packing viewed down the c axis showing an edge view of one corrugated sheet with hydrogen bonds drawn as red and blue dashed lines.

**Fig. 3.**
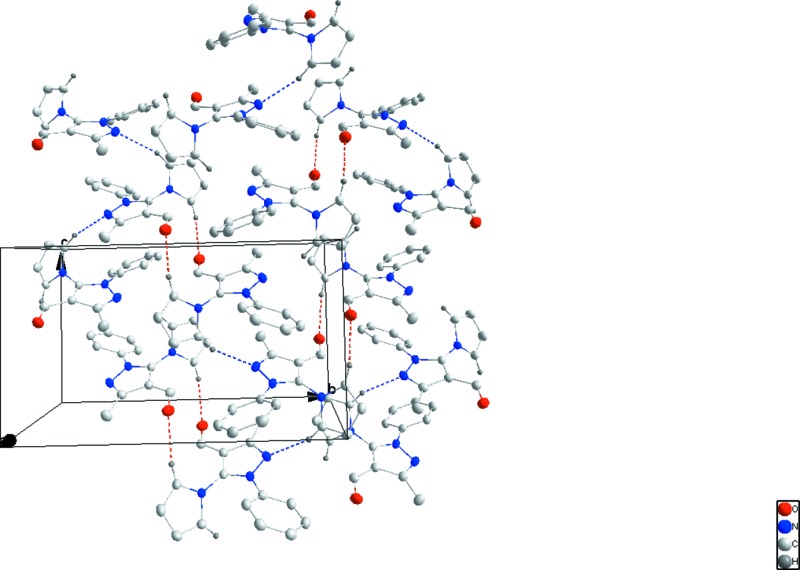
Packing viewed down the a axis showing the C—H···O and C—H···N interactions as red and blue dashed lines, respectively.

. DOI: 10.1107/S1600536814020984/sj5428fig1.tif
Perspective view of the title mol­ecule with labeling scheme and 50% probability ellipsoids for non-H atoms.

c . DOI: 10.1107/S1600536814020984/sj5428fig2.tif
Packing viewed down the *c* axis showing an edge view of one corrugated sheet with hydrogen bonds drawn as red and blue dashed lines.

a . DOI: 10.1107/S1600536814020984/sj5428fig3.tif
Packing viewed down the *a* axis showing the C—H⋯O and C—H⋯N inter­actions as red and blue dashed lines, respectively.

CCDC reference: 1025251

Additional supporting information:  crystallographic information; 3D view; checkCIF report

## Figures and Tables

**Table 1:** Hydrogen-bond geometry (Å, °)

*D*—H⋯*A*	*D*—H	H⋯*A*	*D*⋯*A*	*D*—H⋯*A*
C15—H15⋯N1^i^	0.95	2.48	3.3931 (17)	161
C12—H12⋯O1^ii^	0.95	2.52	3.4255 (17)	159

Symmetry codes: (i) 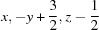; (ii) 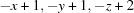.

## supplementary crystallographic information

### S1. Comment

Pyrazolines and substituted pyrazolines exhibit a variety of biological activities displaying anti-bacterial (Nauduri & Reddy, 1998), anti-fungal (Korgaokar *et al.*, 1996), anti-tumor (Taylor & Patel, 1992), anticonvulsant (Ozdemir *et al.*, 2007; Ruhoğlu *et al.*, 2005), anti-depressant (Palaska *et al.*, 2001; Rajendra Prasad *et al.*, 2005) and anti-inflammatory (Udupi *et al.*, 1998) properties. Moreover, pyrazolo[3,4-*b*]pyrazines are also used as fluorescent and disperse dyes in dye chemistry (Rangnekar & Dhamnaskar, 1990; Kopp *et al.*, 2001). In addition the title compound and its analogs have proved to be versatile compounds for use in the synthesis of several heterocycles (Farghaly & El-Kashef, 2005). Based on these findings and as part of our on-going study of the synthesis of bio-heterocyclic molecules, we report in this study the crystal structure of the title compound.

In the title compound (Fig. 1), the central pyrazole ring makes dihedral angles of 58.99 (5) and 34.95 (5)°, respectively, with the the pyrrolyl and phenyl rings. Weak, pairwise C12—H12···O1 interactions across centers of symmetry form dimers which are further associated into corrugated sheets running approximately parallel to (100) *via* weak C15—H15···N1 interactions (Table 1, Fig. 2 and Fig. 3).

### S2. Experimental

A mixture of 2.01 g (0.01 mol) 5-amino-3-methyl-1-phenyl-1*H*-pyrazole-4-carbaldehyde and 1.32 g (0.01 mol) of 2,5-dimethoxytetrahydrofuran in acetic acid (15 ml) was heated under reflux for 2 h. After cooling the mixture was poured into cold water (50 ml) and the precipitate was filtered off, washed with water, dried under vacuum and crystallized from dioxane-water (3:1vv) to afford the product in 85% yield. Colourless plate-like crystals for X-ray diffraction were obtained by further crystallization of the product from acetic acid. *M*.p. 409 – 411 K.

### S3. Refinement

H-atoms attached to carbon were placed in calculated positions (C—H = 0.95 - 0.98 Å). All were included as riding contributions with isotropic displacement parameters 1.2 - 1.5 times those of the attached atoms.

### Crystal data

**Table:**

C_15_H_13_N_3_O	*F*(000) = 528
*M**_r_* = 251.28	*D*_x_ = 1.317 Mg m^−^^3^
Monoclinic, *P*2_1_/*c*	Mo *K*α radiation, λ = 0.71073 Å
*a* = 9.5807 (8) Å	Cell parameters from 6992 reflections
*b* = 15.1720 (13) Å	θ = 2.5–29.1°
*c* = 8.7370 (8) Å	µ = 0.09 mm^−^^1^
β = 93.6180 (11)°	*T* = 150 K
*V* = 1267.46 (19) Å^3^	Plate, colourless
*Z* = 4	0.29 × 0.17 × 0.04 mm

### Data collection

**Table:**

Bruker SMART APEX CCD diffractometer	3321 independent reflections
Radiation source: fine-focus sealed tube	2527 reflections with *I* > 2σ(*I*)
Graphite monochromator	*R*_int_ = 0.053
Detector resolution: 8.3660 pixels mm^-1^	θ_max_ = 29.2°, θ_min_ = 2.1°
φ and ω scans	*h* = −13→13
Absorption correction: multi-scan (*SADABS*; Bruker, 2014)	*k* = −20→20
*T*_min_ = 0.98, *T*_max_ = 1.00	*l* = −11→11
26282 measured reflections	

### Refinement

**Table:**

Refinement on *F*^2^	Primary atom site location: structure-invariant direct methods
Least-squares matrix: full	Secondary atom site location: difference Fourier map
*R*[*F*^2^ > 2σ(*F*^2^)] = 0.044	Hydrogen site location: inferred from neighbouring sites
*wR*(*F*^2^) = 0.114	H-atom parameters constrained
*S* = 1.07	*w* = 1/[σ^2^(*F*_o_^2^) + (0.0533*P*)^2^ + 0.2193*P*] where *P* = (*F*_o_^2^ + 2*F*_c_^2^)/3
3321 reflections	(Δ/σ)_max_ < 0.001
173 parameters	Δρ_max_ = 0.31 e Å^−^^3^
0 restraints	Δρ_min_ = −0.25 e Å^−^^3^

### Special details

**Table:**

Experimental. The diffraction data were collected in three sets of400 frames (0.5° width in ω) at φ = 0, 120and 240°. A scan time of 80 sec/frame was used.
Geometry. All e.s.d.'s (except the e.s.d. in the dihedral angle between two l.s. planes)are estimated using the full covariance matrix. The cell e.s.d.'s are takeninto account individually in the estimation of e.s.d.'s in distances, anglesand torsion angles; correlations between e.s.d.'s in cell parameters are onlyused when they are defined by crystal symmetry. An approximate (isotropic)treatment of cell e.s.d.'s is used for estimating e.s.d.'s involving l.s. planes.
Refinement. Refinement of *F*^2^ against ALL reflections. The weighted *R*-factor *wR* andgoodness of fit *S* are based on *F*^2^, conventional *R*-factors *R* are basedon *F*, with *F* set to zero for negative *F*^2^. The threshold expression of*F*^2^ > σ(*F*^2^) is used only for calculating *R*-factors(gt) *etc*. and isnot relevant to the choice of reflections for refinement. *R*-factors basedon *F*^2^ are statistically about twice as large as those based on *F*, and *R*-factors based on ALL data will be even larger. H-atoms attached to carbonwere placed in calculated positions (C—H = 0.95 - 0.98 Å). All wereincluded as riding contributions with isotropic displacement parameters1.2 - 1.5 times those of the attached atoms.

### Fractional atomic coordinates and isotropic or equivalent isotropic displacement parameters (Å^2^)

**Table:**

	*x*	*y*	*z*	*U*_iso_*/*U*_eq_	
O1	0.33317 (10)	0.54403 (7)	0.91467 (11)	0.0336 (2)	
N1	0.59037 (11)	0.76421 (7)	0.80193 (12)	0.0234 (2)	
N2	0.67710 (11)	0.71108 (7)	0.72255 (12)	0.0211 (2)	
N3	0.70035 (11)	0.55911 (6)	0.64732 (12)	0.0210 (2)	
C1	0.63304 (13)	0.62637 (8)	0.72276 (14)	0.0208 (3)	
C2	0.51547 (13)	0.62272 (8)	0.80704 (14)	0.0217 (3)	
C3	0.49451 (13)	0.71148 (8)	0.85367 (14)	0.0230 (3)	
C4	0.38179 (14)	0.74728 (10)	0.94605 (16)	0.0306 (3)	
H4A	0.3977	0.8103	0.9644	0.046*	
H4B	0.3827	0.7162	1.0445	0.046*	
H4C	0.2909	0.7388	0.8901	0.046*	
C5	0.43357 (14)	0.54534 (9)	0.83563 (15)	0.0253 (3)	
H5	0.4597	0.4916	0.7896	0.030*	
C6	0.79899 (13)	0.74965 (8)	0.66330 (14)	0.0206 (3)	
C7	0.92330 (13)	0.70303 (8)	0.66282 (14)	0.0242 (3)	
H7	0.9285	0.6441	0.6997	0.029*	
C8	1.03977 (14)	0.74327 (9)	0.60798 (15)	0.0269 (3)	
H8	1.1251	0.7115	0.6063	0.032*	
C9	1.03304 (14)	0.82956 (9)	0.55550 (15)	0.0276 (3)	
H9	1.1131	0.8567	0.5172	0.033*	
C10	0.90846 (15)	0.87586 (9)	0.55948 (15)	0.0276 (3)	
H10	0.9040	0.9354	0.5256	0.033*	
C11	0.79111 (13)	0.83644 (8)	0.61199 (15)	0.0240 (3)	
H11	0.7057	0.8682	0.6132	0.029*	
C12	0.76608 (14)	0.48753 (8)	0.71948 (15)	0.0250 (3)	
H12	0.7625	0.4720	0.8245	0.030*	
C13	0.83647 (15)	0.44366 (9)	0.61277 (15)	0.0288 (3)	
H13	0.8913	0.3920	0.6299	0.035*	
C14	0.81336 (14)	0.48874 (9)	0.47146 (15)	0.0276 (3)	
H14	0.8493	0.4722	0.3767	0.033*	
C15	0.73103 (14)	0.55959 (8)	0.49470 (14)	0.0240 (3)	
H15	0.7001	0.6018	0.4198	0.029*	

### Atomic displacement parameters (Å^2^)

**Table:**

	*U*^11^	*U*^22^	*U*^33^	*U*^12^	*U*^13^	*U*^23^
O1	0.0322 (5)	0.0361 (6)	0.0335 (5)	−0.0085 (4)	0.0099 (4)	0.0015 (4)
N1	0.0209 (5)	0.0216 (5)	0.0282 (6)	0.0022 (4)	0.0048 (4)	−0.0046 (4)
N2	0.0209 (5)	0.0177 (5)	0.0251 (5)	0.0001 (4)	0.0044 (4)	−0.0023 (4)
N3	0.0249 (5)	0.0171 (5)	0.0214 (5)	0.0011 (4)	0.0038 (4)	−0.0007 (4)
C1	0.0233 (6)	0.0172 (6)	0.0216 (6)	−0.0004 (5)	0.0002 (5)	−0.0002 (4)
C2	0.0217 (6)	0.0222 (6)	0.0211 (6)	−0.0005 (5)	0.0012 (5)	−0.0007 (5)
C3	0.0214 (6)	0.0242 (6)	0.0232 (6)	0.0006 (5)	0.0007 (5)	−0.0024 (5)
C4	0.0258 (7)	0.0327 (7)	0.0341 (7)	0.0010 (6)	0.0071 (6)	−0.0065 (6)
C5	0.0274 (6)	0.0254 (6)	0.0229 (6)	−0.0033 (5)	0.0008 (5)	0.0006 (5)
C6	0.0218 (6)	0.0197 (6)	0.0204 (6)	−0.0025 (5)	0.0033 (5)	−0.0022 (5)
C7	0.0249 (6)	0.0208 (6)	0.0270 (6)	0.0007 (5)	0.0028 (5)	−0.0009 (5)
C8	0.0234 (6)	0.0277 (7)	0.0299 (7)	−0.0003 (5)	0.0044 (5)	−0.0049 (5)
C9	0.0279 (7)	0.0279 (7)	0.0275 (7)	−0.0077 (5)	0.0067 (5)	−0.0042 (5)
C10	0.0331 (7)	0.0214 (6)	0.0284 (7)	−0.0041 (5)	0.0026 (5)	0.0005 (5)
C11	0.0253 (6)	0.0192 (6)	0.0272 (6)	0.0010 (5)	0.0005 (5)	−0.0011 (5)
C12	0.0315 (7)	0.0184 (6)	0.0250 (6)	0.0016 (5)	0.0002 (5)	0.0021 (5)
C13	0.0342 (7)	0.0209 (6)	0.0313 (7)	0.0062 (5)	0.0012 (6)	−0.0015 (5)
C14	0.0318 (7)	0.0276 (7)	0.0238 (6)	0.0024 (5)	0.0043 (5)	−0.0041 (5)
C15	0.0294 (7)	0.0234 (6)	0.0195 (6)	0.0012 (5)	0.0028 (5)	0.0002 (5)

### Geometric parameters (Å, º)

**Table:**

O1—C5	1.2191 (16)	C6—C11	1.3915 (17)
N1—C3	1.3191 (16)	C7—C8	1.3838 (18)
N1—N2	1.3762 (14)	C7—H7	0.9500
N2—C1	1.3529 (15)	C8—C9	1.3873 (19)
N2—C6	1.4319 (15)	C8—H8	0.9500
N3—C15	1.3835 (16)	C9—C10	1.3873 (19)
N3—C12	1.3869 (16)	C9—H9	0.9500
N3—C1	1.3946 (15)	C10—C11	1.3775 (18)
C1—C2	1.3852 (17)	C10—H10	0.9500
C2—C3	1.4248 (17)	C11—H11	0.9500
C2—C5	1.4427 (17)	C12—C13	1.3589 (19)
C3—C4	1.4909 (17)	C12—H12	0.9500
C4—H4A	0.9800	C13—C14	1.4165 (19)
C4—H4B	0.9800	C13—H13	0.9500
C4—H4C	0.9800	C14—C15	1.3562 (18)
C5—H5	0.9500	C14—H14	0.9500
C6—C7	1.3854 (17)	C15—H15	0.9500
			
C3—N1—N2	105.85 (10)	C8—C7—C6	119.21 (12)
C1—N2—N1	110.94 (10)	C8—C7—H7	120.4
C1—N2—C6	130.57 (10)	C6—C7—H7	120.4
N1—N2—C6	118.36 (10)	C7—C8—C9	120.60 (12)
C15—N3—C12	108.92 (10)	C7—C8—H8	119.7
C15—N3—C1	125.76 (10)	C9—C8—H8	119.7
C12—N3—C1	124.63 (10)	C10—C9—C8	119.45 (12)
N2—C1—C2	107.63 (10)	C10—C9—H9	120.3
N2—C1—N3	122.74 (11)	C8—C9—H9	120.3
C2—C1—N3	129.63 (11)	C11—C10—C9	120.67 (12)
C1—C2—C3	104.38 (11)	C11—C10—H10	119.7
C1—C2—C5	126.44 (11)	C9—C10—H10	119.7
C3—C2—C5	129.17 (12)	C10—C11—C6	119.30 (12)
N1—C3—C2	111.19 (11)	C10—C11—H11	120.4
N1—C3—C4	120.56 (11)	C6—C11—H11	120.4
C2—C3—C4	128.25 (12)	C13—C12—N3	107.60 (11)
C3—C4—H4A	109.5	C13—C12—H12	126.2
C3—C4—H4B	109.5	N3—C12—H12	126.2
H4A—C4—H4B	109.5	C12—C13—C14	107.73 (12)
C3—C4—H4C	109.5	C12—C13—H13	126.1
H4A—C4—H4C	109.5	C14—C13—H13	126.1
H4B—C4—H4C	109.5	C15—C14—C13	108.21 (12)
O1—C5—C2	124.65 (12)	C15—C14—H14	125.9
O1—C5—H5	117.7	C13—C14—H14	125.9
C2—C5—H5	117.7	C14—C15—N3	107.53 (11)
C7—C6—C11	120.75 (11)	C14—C15—H15	126.2
C7—C6—N2	120.88 (11)	N3—C15—H15	126.2
C11—C6—N2	118.31 (11)		
			
C3—N1—N2—C1	1.28 (13)	C3—C2—C5—O1	−4.1 (2)
C3—N1—N2—C6	−174.98 (10)	C1—N2—C6—C7	−32.38 (19)
N1—N2—C1—C2	−1.18 (13)	N1—N2—C6—C7	143.01 (12)
C6—N2—C1—C2	174.49 (11)	C1—N2—C6—C11	150.22 (13)
N1—N2—C1—N3	178.45 (10)	N1—N2—C6—C11	−34.39 (16)
C6—N2—C1—N3	−5.88 (19)	C11—C6—C7—C8	−1.13 (19)
C15—N3—C1—N2	−53.33 (18)	N2—C6—C7—C8	−178.47 (11)
C12—N3—C1—N2	116.10 (14)	C6—C7—C8—C9	0.68 (19)
C15—N3—C1—C2	126.21 (14)	C7—C8—C9—C10	0.54 (19)
C12—N3—C1—C2	−64.36 (19)	C8—C9—C10—C11	−1.3 (2)
N2—C1—C2—C3	0.59 (13)	C9—C10—C11—C6	0.87 (19)
N3—C1—C2—C3	−179.00 (12)	C7—C6—C11—C10	0.37 (19)
N2—C1—C2—C5	179.33 (11)	N2—C6—C11—C10	177.77 (11)
N3—C1—C2—C5	−0.3 (2)	C15—N3—C12—C13	−0.26 (15)
N2—N1—C3—C2	−0.88 (14)	C1—N3—C12—C13	−171.20 (11)
N2—N1—C3—C4	−179.99 (11)	N3—C12—C13—C14	−0.27 (15)
C1—C2—C3—N1	0.20 (14)	C12—C13—C14—C15	0.71 (16)
C5—C2—C3—N1	−178.50 (12)	C13—C14—C15—N3	−0.86 (15)
C1—C2—C3—C4	179.21 (12)	C12—N3—C15—C14	0.70 (15)
C5—C2—C3—C4	0.5 (2)	C1—N3—C15—C14	171.52 (12)
C1—C2—C5—O1	177.49 (13)		

### Hydrogen-bond geometry (Å, º)

**Table:**

*D*—H···*A*	*D*—H	H···*A*	*D*···*A*	*D*—H···*A*
C15—H15···N1^i^	0.95	2.48	3.3931 (17)	161
C12—H12···O1^ii^	0.95	2.52	3.4255 (17)	159

Symmetry codes: (i) *x*, −*y*+3/2, *z*−1/2; (ii) −*x*+1, −*y*+1, −*z*+2.
