# Supplementary figures and images for: Crystal structure of 3-methyl-1-phenyl-5-(1H-pyrrol-1-yl)-1H-pyrazole-4-carbaldehyde
Source: Acta Crystallogr Sect E Struct Rep Online. 2014 Sep 27;70(Pt 10):o1131–2. doi: 10.1107/S1600536814020984 (PMC4257204; doi:10.1107/S1600536814020984)

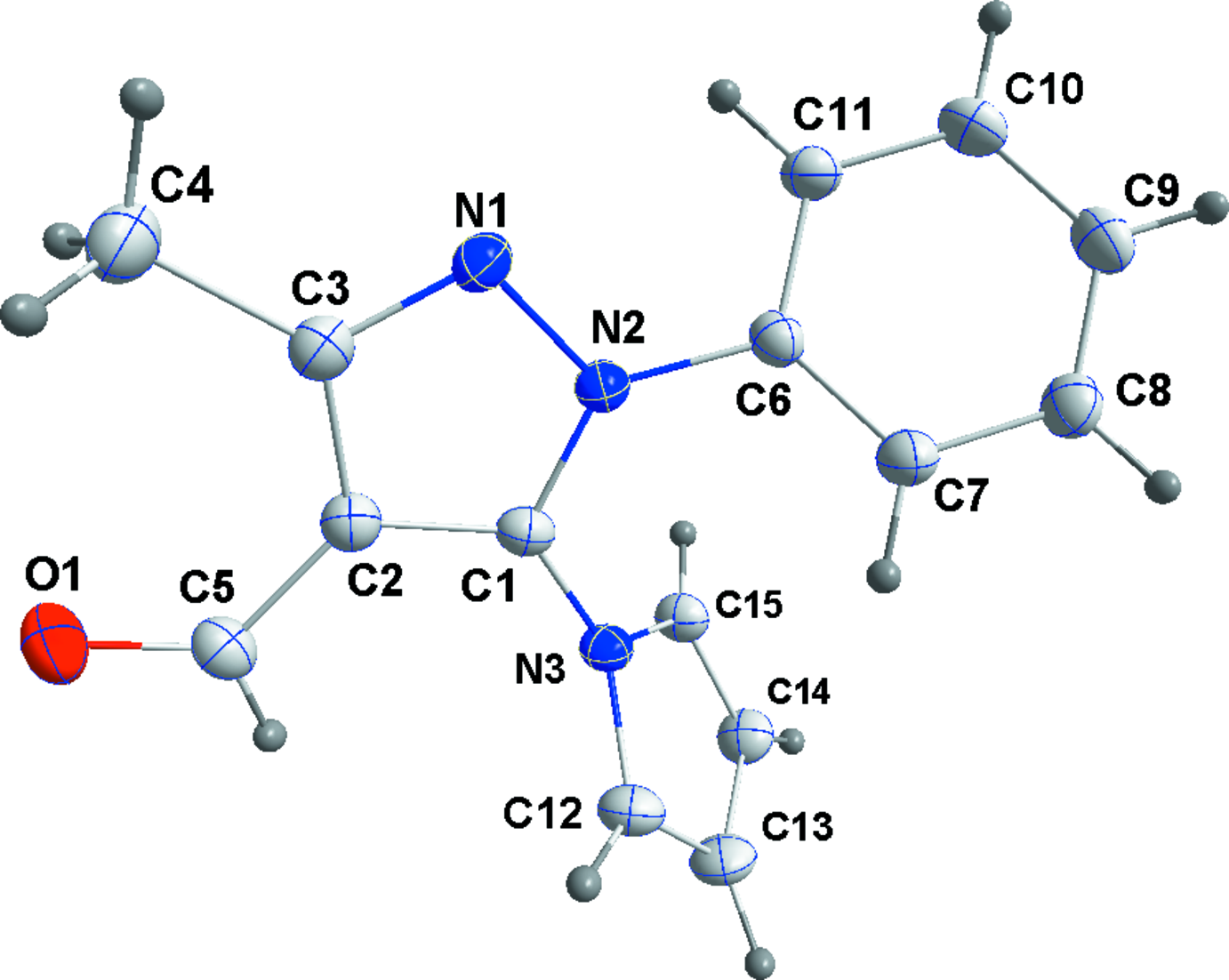

Supplement: Supplementary file 4 [file e-70-o1131-fig1.tif]

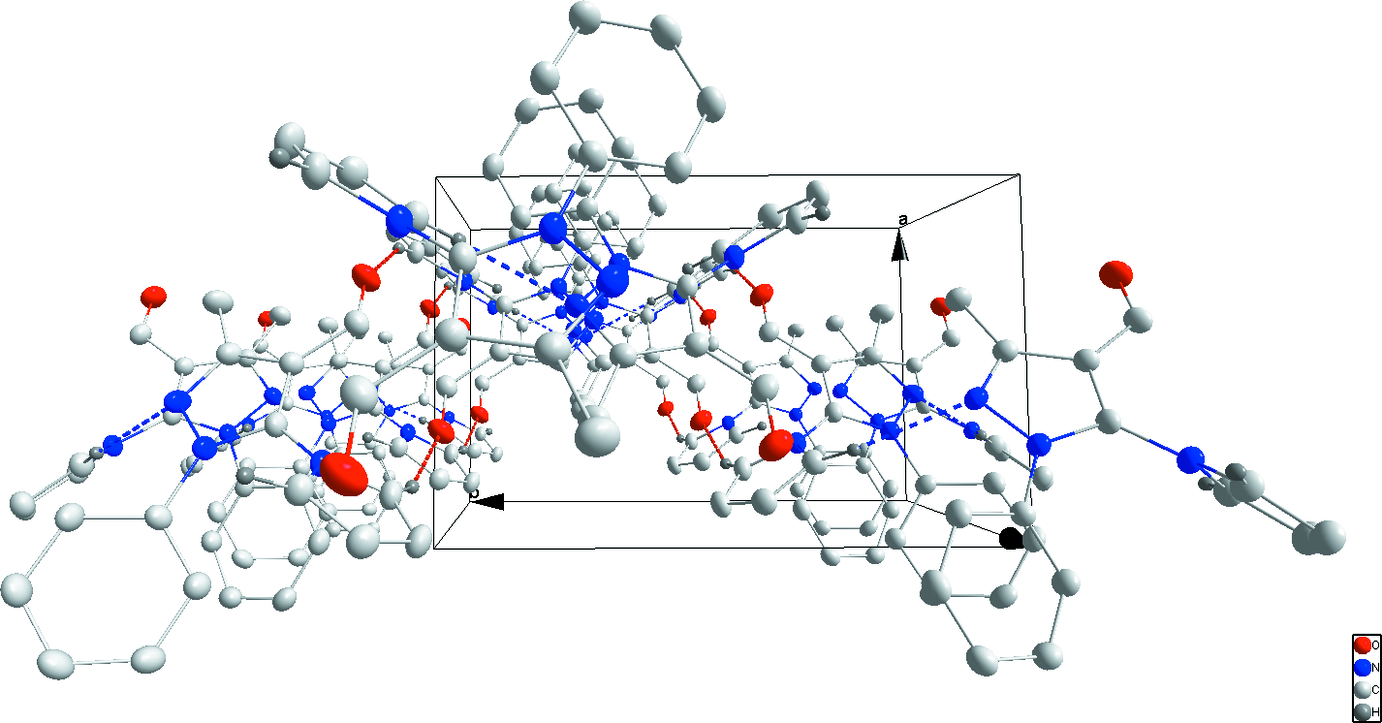

Supplement: Supplementary file 5 [file e-70-o1131-fig2.tif]

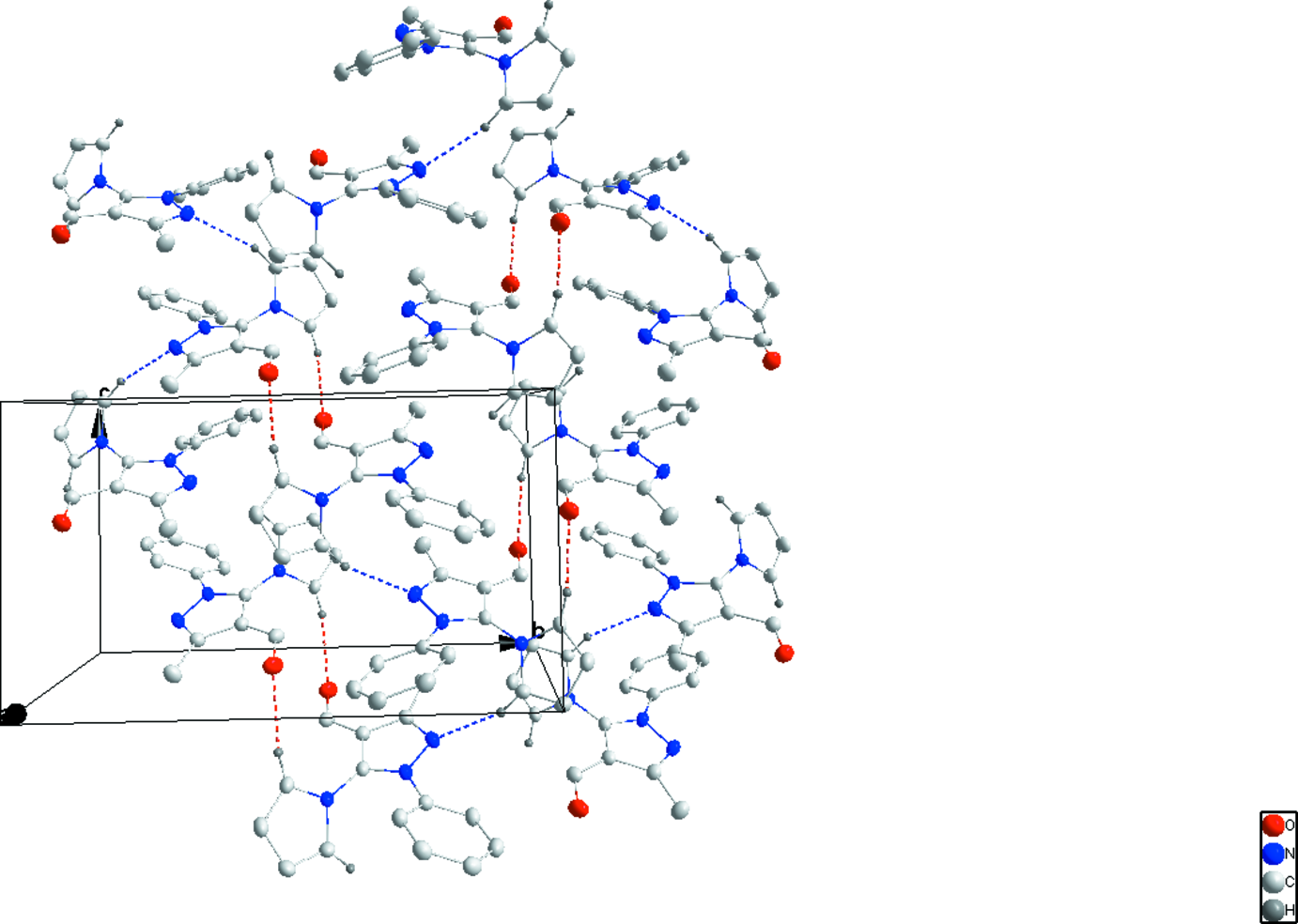

Supplement: Supplementary file 6 [file e-70-o1131-fig3.tif]
